# WSB2 inhibits apoptosis and autophagy by targeting NOXA for degradation

**DOI:** 10.1002/mco2.70071

**Published:** 2025-01-24

**Authors:** Shengpeng Shao, Danrui Cui, Chutian Zheng, Xiufang Xiong, Yongchao Zhao

**Affiliations:** ^1^ Department of Hepatobiliary and Pancreatic Surgery The First Affiliated Hospital and Institute of Translational Medicine Zhejiang University School of Medicine Hangzhou China; ^2^ Cancer Institute of the Second Affiliated Hospital and Institute of Translational Medicine Zhejiang University School of Medicine Hangzhou China

**Keywords:** CUL5, degradation, NOXA, ubiquitylation, WSB2

1

NOXA protein, a pro‐apoptotic member of the BCL2 (B‐cell lymphoma 2) protein family, exhibits a high affinity for binding to MCL1 (myeloid cell leukemia 1) and interacts with BCL2A1 (B‐cell lymphoma 2‐related protein A1). These interactions release BIM (BCL2 like protein 11), triggering apoptosis. Furthermore, NOXA facilitates proteasome‐mediated MCL1 degradation, a critical response to various anti‐cancer drugs and extracellular stimuli, including UV (ultraviolet) irradiation. Additionally, during oncogenic RAS activation, NOXA induces autophagic cell death by displacing MCL1 from Beclin‐1, a key component of the class III PI3K (phosphoinositide 3‐kinase) complex required for autophagosome biogenesis.^1^ Therefore, NOXA plays an essential role in regulating apoptotic and autophagic cell death, and its induction is a promising therapeutic target for anti‐cancer treatments.^2^

NOXA level regulation is tightly controlled at both transcriptional and post‐translational levels. Previous studies have demonstrated that CRL5 (Cullin‐RING Ligase 5) mediates NOXA ubiquitylation and degradation.^3^ The CRL5 complex comprises four components: a scaffold protein (CUL5), adaptor proteins (Elongin B/C), a substrate receptor SOCS (suppressor of cytokine signaling) protein, and a RING protein (RBX2/SAG). The SOCS protein specifically recognizes and binds to substrates.^3^ In mammalian cells, there are 37 SOCS substrate receptor proteins.^3^ However, the specific receptors for NOXA recognition remain unidentified, posing a challenge to the development of inhibitors targeting NOXA degradation. In this study, we demonstrate that the substrate receptor protein WSB2 (WD repeat and SOCS box containing 2) targets NOXA for degradation.

To identify the receptor in the CRL5 complex responsible for recognizing and binding NOXA, we performed an siRNA‐based screening targeting all known CRL5 receptor proteins and HSP90A/B in Huh7 cells. HSP90A/B client proteins undergo degradation by the CRL5 ligase following treatment with HSP90 inhibitors. Subsequently, NOXA accumulation was assessed via immunoblotting. WSB2 emerged as a candidate due to the highest accumulation of NOXA observed upon its knockdown (Figure S1A). Next, we observed a significant increase in NOXA protein levels upon WSB2 silencing across various cancer cell lines, including Huh7, H1299, and A549 (Figure 1A). Interestingly, WSB2 knockdown did not alter NOXA mRNA levels in Huh7 and H1299 cells but moderately increased them in A549 cells (Figure 1A). The observed increase in NOXA mRNA levels in A549 cells, which harbor wild‐type p53, is likely due to transcriptional activation of p53. Since p53 is a known substrate of WSB2,^4^ its accumulation following WSB2 knockdown could explain this transcriptional effect. These findings suggest that WSB2 primarily regulates NOXA levels at the post‐translational level. Consistent with this, MG132, a proteasome inhibitor, effectively prevented WSB2 overexpression‐induced NOXA degradation in both Huh7 and H1299 cells (Figure S1B). In contrast, CQ (chloroquine), a lysosome inhibitor, inhibited NOXA degradation in Huh7 cells but unexpectedly reduced NOXA in H1299 cells (Figure S1B). These results suggest that WSB2 primarily promotes NOXA degradation via the UPS (ubiquitin‐proteasome system). Furthermore, WSB2 silencing significantly extended the half‐life of the NOXA protein in Huh7 and H1299 cells, indicating that WSB2 regulates NOXA protein stability (Figure 1A). Additionally, endogenous NOXA was readily detected in FLAG‐WSB2 immunoprecipitates, confirming an interaction between WSB2 and NOXA (Figure S1B).

**FIGURE 1:**
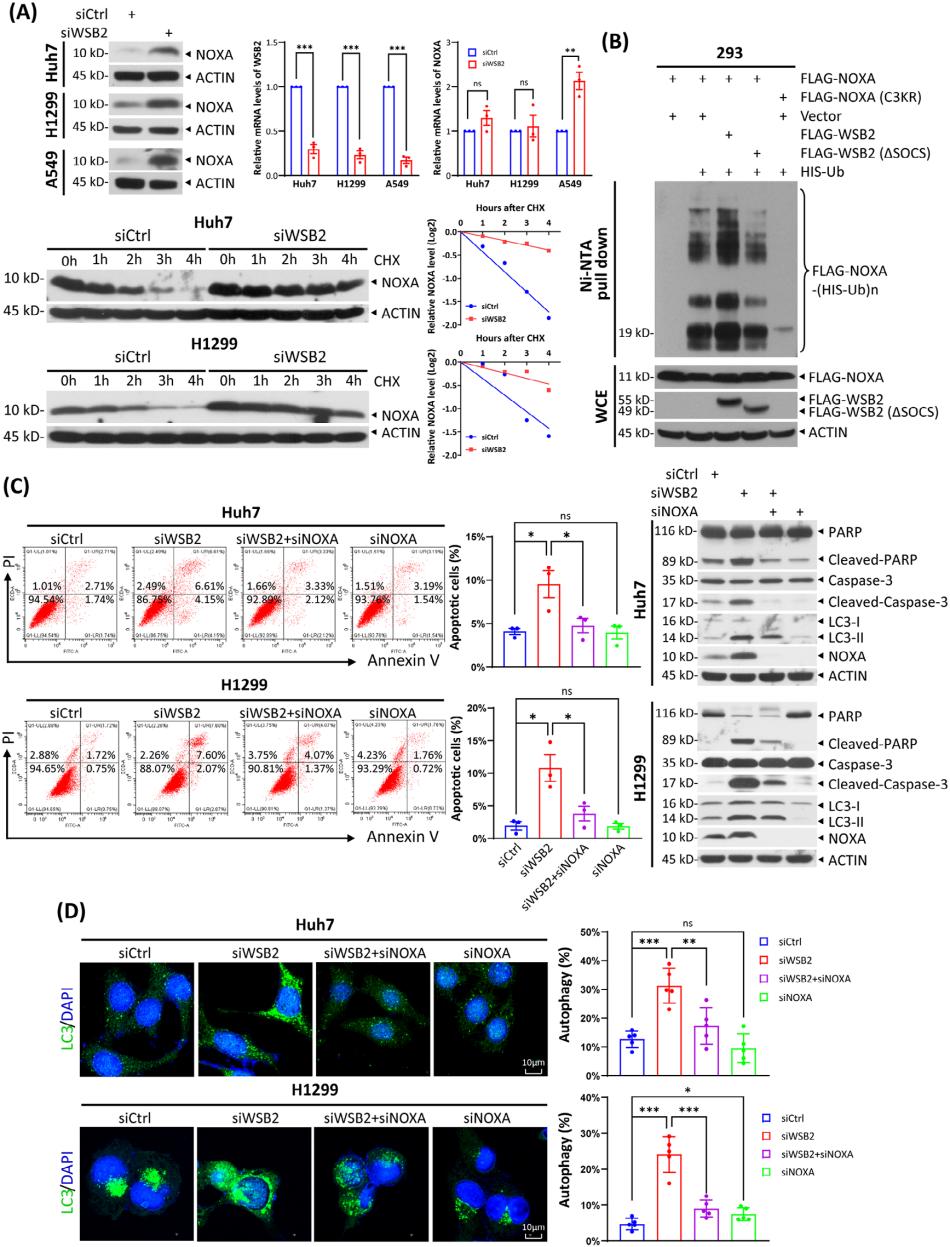
**CRL5^WSB2^ targets NOXA for degradation to inhibit apoptosis and autophagy**. (A) Cells were transfected with the indicated siRNAs for 48 h, and then subjected to immunoblotting (IB) with the indicated antibodies (Abs) or qRT‐PCR analysis (top), or treated with 100 µg/mL cycloheximide (CHX) for the indicated time periods, followed by IB with the indicated Abs (bottom). Data from three independent experiments were expressed as mean ± SEM, **p < 0.01, ***p < 0.001, ns: not significant. Densitometry quantification was performed with ImageJ, and the decay curves are shown (bottom right). (B) HEK293 cells transfected with the indicated plasmids for 48 h were treated with 20 µM MG132 for 6 h, lysed under denaturing conditions, and then subjected to a pull‐down assay using Ni‐NTA beads. Pull‐downs (top) and whole cell extracts (bottom) were subjected to IB with the indicated Abs. (C) Huh7 and H1299 cells were transfected with the indicated siRNAs for 48 h, followed by IB with the indicated Abs (right) or FACS analysis to determine the apoptotic cells (left, a representative FACS profile, and the percentage of annexin V+ cells, mean ± SEM; *n* = 3; ns: not significant, *p < 0.05). (D) Huh7 and H1299 cells transfected with the indicated siRNAs for 48 h were stained with LC3B Ab (green) and DAPI (blue), and photographed under a confocal fluorescence microscope (left). The percentage of cells with punctate structures of LC3B represents the proportion of cells undergoing autophagy (right) (mean ± SD, *n* = 5, *p < 0.05, **p < 0.01, ***p < 0.001).

As proteins targeted for degradation via the UPS require polyubiquitylation, we investigated whether WSB2 mediates NOXA polyubiquitylation through an in vivo ubiquitylation assay. The results demonstrated that wild‐type WSB2 significantly enhanced the polyubiquitylation of ectopically expressed NOXA. In contrast, WSB2‐∆SOCS, which encodes a WSB2 protein lacking the SOCS box—a domain required for binding Elongin B/C and CUL5—had no such effect, indicating that WSB2 promotes NOXA polyubiquitylation in a CRL5‐dependent manner (Figure 1B). As a negative control, a NOXA mutant (C3KR), in which Lys35, Lys41, and Lys48 were simultaneously mutated to arginine residues,^5^ exhibited a complete loss of ubiquitylation (Figure 1B). Collectively, these findings demonstrate that WSB2 targets NOXA for polyubiquitylation and subsequent degradation via the UPS.

Given that NOXA plays a crucial role in regulating both apoptotic and autophagic cell death, we investigated whether NOXA accumulation resulting from WSB2 inactivation significantly contributes to the induction of apoptosis and autophagy. The increase in cleaved PARP (poly‐ADP‐ribose polymerase) and cleaved caspase‐3 levels, two well‐established apoptosis markers, observed following WSB2 knockdown in Huh7 and H1299 cells, was abolished when NOXA was simultaneously silenced (Figure 1C). Similarly, the increased percentage of apoptotic cells marked by annexin V+ induced by WSB2 silencing was reversed upon simultaneous NOXA knockdown (Figure 1C).

Moreover, WSB2 silencing induced the conversion of LC3‐I to LC3‐II, a well‐known marker of autophagy induction, indicating that WSB2 silencing promotes autophagy (Figure 1C). Simultaneous NOXA knockdown partially reversed this conversion, demonstrating that NOXA accumulation plays a causal role in autophagy induction (Figure 1C). Likewise, LC3‐positive puncta formation, another hallmark of autophagy, was significantly induced by WSB2 silencing, and this effect was reversed by simultaneous NOXA knockdown (Figure 1D). Moreover, the autophagic flux induced by WSB2 knockdown was disrupted by Baf A1 (bafilomycin A1), a vacuolar H^+^‐ATPase inhibitor that impedes lysosomal acidification and protein degradation. This disruption was evidenced by the accumulation of both LC3‐I and LC3‐II and increased LC3‐positive puncta formation (Figure S1C). Mechanistically, WSB2 knockdown and subsequent NOXA accumulation enhanced the binding of NOXA to MCL1, thereby releasing more Beclin‐1 to facilitate the formation of the class III PI3K complex (Figure S1C). This finding is consistent with observations from previous studies.^1^ Together, these results demonstrate that WSB2 negatively regulates apoptosis and autophagy in a NOXA‐dependent manner.

Unlike apoptosis, which directly eliminates tumor cells, autophagy can support tumor cell survival as well as promote cell death. To determine the role of autophagy induced by WSB2 inactivation in cell survival and death, we knocked down ATG5 to impair autophagy following WSB2 silencing and evaluated cell growth using the CCK8 assay and cell survival using the clonogenic assay. WSB2 silencing significantly suppressed cell growth and survival, whereas simultaneous ATG5 knockdown partially rescued these effects (Figure S1D). These findings suggest that WSB2 depletion induces cytotoxic autophagy.

Collectively, our study demonstrates that CRL5^WSB2^ targets NOXA for ubiquitylation and subsequent proteasomal degradation, thereby inhibiting apoptosis and autophagic cell death. This finding indicates that the WSB2‐NOXA axis may serve as a promising anti‐cancer target. Considering that CRL5^WSB2^ ligase targets multiple substrates for degradation, developing inhibitors specifically targeting CRL5^WSB2^ ligase is not an optimal strategy for anticancer therapeutics. Instead, the discovery of small molecules disrupting the interaction between WSB2 and NOXA could specifically promote NOXA accumulation, inducing apoptotic and autophagic cell death with minimal off‐target effects. However, several challenges hinder the development of therapeutics targeting the WSB2‐NOXA axis: (1) co‐crystallographic structures of the WSB2‐NOXA complex have not yet been reported; (2) disrupting the interaction between WSB2 and NOXA using small‐molecule compounds is inherently difficult; and (3) since NOXA has no enzymatic activity, only high‐throughput methods for assessing its protein levels can be used, and such methods are relatively limited. Integrating structural biology with computer‐aided virtual screening may provide an effective approach to overcoming these challenges.

## AUTHOR CONTRIBUTIONS

S.S. designed and performed the experiments, analyzed the data, and drafted the manuscript. D.C. designed and performed the experiments, and analyzed the data. C.Z. performed the experiments. X.X. and Y.Z. designed the study, analyzed and interpreted the data, and revised and finalized the manuscript. All authors have reviewed the manuscript.

## CONFLICT OF INTEREST STATEMENT

The authors declare no conflicts of interest.

## Supporting information



Supporting Information

## Data Availability

The authors declare that all data supporting the findings of this study are available with the article or from the corresponding author upon reasonable request.
