# Supplementary material for: WSB2 inhibits apoptosis and autophagy by targeting NOXA for degradation
Source: MedComm (2020). 2025 Jan 24;6(2):e70071. doi: 10.1002/mco2.70071 (PMC11758357; doi:10.1002/mco2.70071)
Supplement: Supplementary file 1 — Supporting Information [file MCO2-6-e70071-s001.docx]

**Supplementary Materials for**

**WSB2 inhibits apoptosis and autophagy by targeting NOXA for degradation**

Shengpeng Shao^1^, Danrui Cui^1^, Chutian Zheng^1^, Xiufang Xiong^2*^

and Yongchao Zhao^1*^

^1^Department of Hepatobiliary and Pancreatic Surgery, the First Affiliated Hospital and Institute of Translational Medicine, Zhejiang University School of Medicine, Hangzhou, China.

^2^Cancer Institute of the Second Affiliated Hospital and Institute of Translational Medicine, Zhejiang University School of Medicine, Hangzhou, China.

*Corresponding authors:

Yongchao Zhao, Department of Hepatobiliary and Pancreatic Surgery, the First Affiliated Hospital, Zhejiang University School of Medicine, 79 Qing-Chun Road, Hangzhou, Zhejiang 310003, China. E-mail: [yongchao@zju.edu.cn](mailto:yongchao@zju.edu.cn)

Xiufang Xiong, Cancer Institute of the Second Affiliated Hospital, Zhejiang University School of Medicine, 268 Kai-Xuan Road, Hangzhou, Zhejiang 310029, China. E-mail: xiufang@zju.edu.cn

**
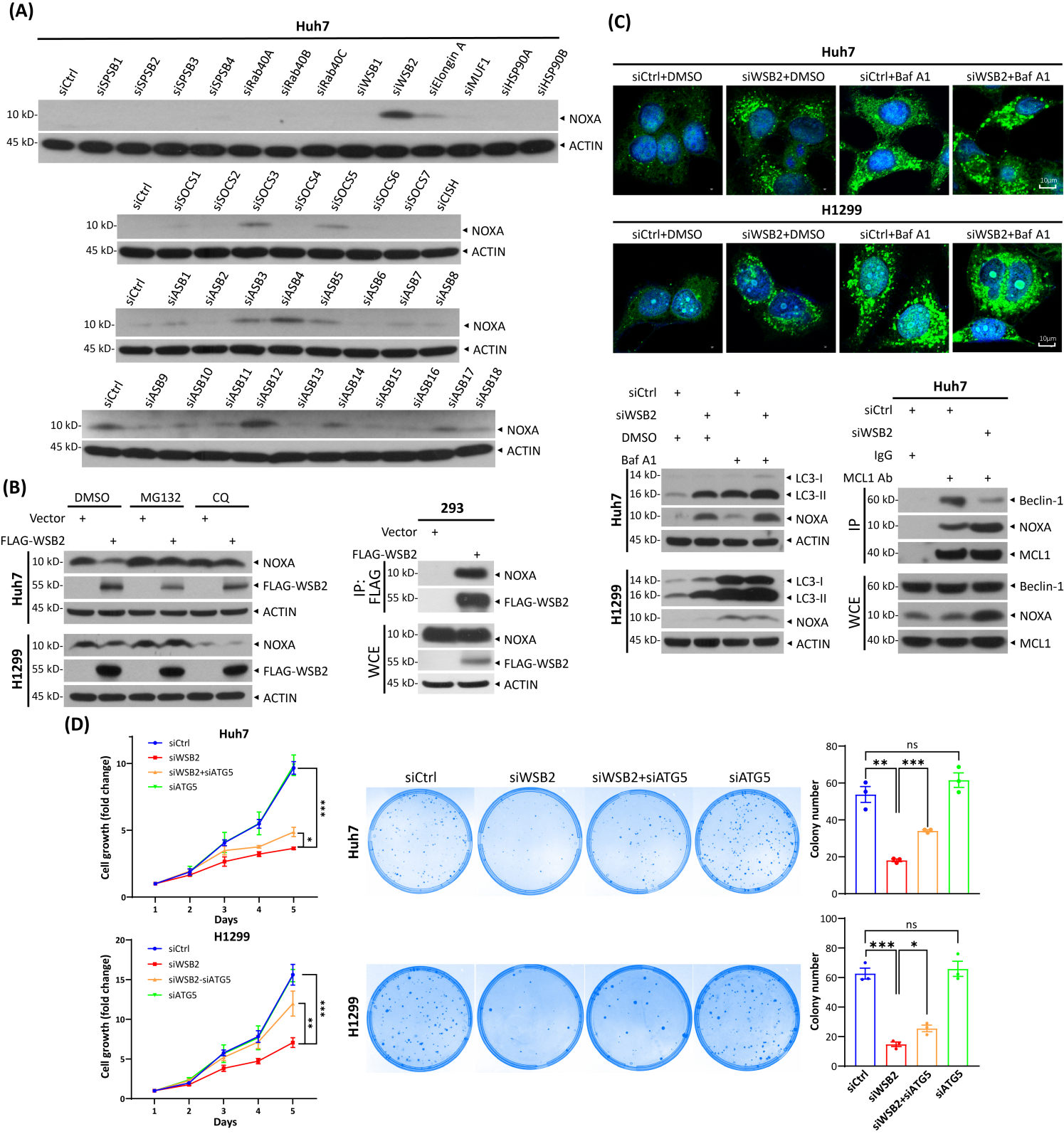
**

**Figure S1. CRL5^WSB2^ targets NOXA for degradation to inhibit apoptosis and autophagy**

(A) Screening the receptor protein in CRL5 complex responsible for NOXA degradation. Huh7 cells were transfected with the indicated siRNAs for 48 h, followed by immunoblotting (IB) with the indicated antibodies (Abs). (B) Huh7 and H1299 cells were transfected with the indicated plasmids for 48 h and then treated with 20 μM MG132 or 50 μM chloroquine (CQ) for 3 h, followed by IB with the indicated Abs (left). HEK293 cells were transfected with mock vector or FLAG-WSB2 plasmids for 48 h and then subjected to immunoprecipitation (IP) with FLAG beads, followed by IB with the indicated Abs (right). WCE: whole cell extracts. (C) Huh7 and H1299 cells were transfected with the indicated siRNAs for 48 h and then treated with 50 nM bafilomycin A1 (Baf A1) for 24 h, followed by immunofluorescent staining with anti-LC3B Ab (up) or IB with the indicated Abs (bottom left). Huh7 were transfected with the indicated siRNAs for 48 h, followed by IP with an anti-MCL1 Ab and then IB with the indicated Abs (bottom right). (D) Huh7 and H1299 cells were transfected with indicated siRNA for 48 h, followed by Cell Counting Kit 8 (CCK8)-based cell growth assay (left), or clonogenic survival assay (right). Mean ± SEM; n = 3; ns, not significant; * *p* < 0.05, ** *p* < 0.01, *** *p* < 0.001.

**Methods and Materials**

**Cell lines and chemicals**

A549, H1299, and HEK293 cells were obtained from American Type Culture Collection (ATCC). A549, HEK293, H1299, and Huh7 cells were maintained in Dulbecco’s modified Eagle’s medium (DMEM) supplemented with 10% (v/v) fetal bovine serum (FBS). All cell lines were authenticated by short tandem repeat (STR) profiling and were confirmed to be free of mycoplasma contamination. The following chemicals were obtained from commercial sources: Bafilomycin A1 (S1413; Selleck), CHX (C7698; Sigma), chloroquine (C6628; Sigma), and MG132 (10012628; Cayman).

**Immunoblotting (IB) and** **immunoprecipitation (IP)**

For IB, whole cells were harvested and lysed in lysis buffer [50mM Tris-HCl (pH 7.5), 0.15M NaCl, 1% NP-40, 0.1% SDS, 1mM EDTA, 1mM DTT, 0.5% Sodium Deoxycholate, 50mM NaF, 1mM Na_3_VO_4_] with protease (11836170001; Roche) and phosphatase inhibitors (11697498001; Roche). Protein concentration was measured using the BCA protein assay kit (23225; Thermo). Equal amounts of lysates were subjected to IB. For IP, cell lysates were incubated with FLAG antibody-conjugated beads (A2220; Sigma) or MCL-1 antibody followed by protein-G beads for 6 hours. The immunoprecipitates were then washed four times with lysis buffer and subjected to IB.

The following antibodies were used: NOXA (OP180; Calbiochem; 1:1000), Beclin-1(3495; Cell Signaling Technology; 1:1000), FLAG (F1804; Sigma; 1:2000), FLAG (F7425; Sigma; 1:5000), MCL1(16225-1-AP; proteintech; 1:1000), PARP (9542; Cell Signaling Technology; 1:1000), caspase-3 (9665; Cell Signaling Technology; 1:1000), LC3B (2775; Cell Signaling Technology; 1:4000), and ACTIN (A5441; Sigma; 1:10000).

**Quantitative RT-PCR (qRT-PCR)**

Total RNA was isolated from cells using TRIzol reagent (15596018; Invitrogen). cDNA was made from RNA using the PrimeScript RT reagent kit (RR037A; Takara). RT-qPCR was performed using SYBR® Premix Ex Taq™ (RR420A; Takara) on the CFX96 Real-time PCR System (Bio-Rad). The primers used for qRT-PCR were as follows: NOXA forward: 5′-CGG GCT CCA GCA GAG CT-3′, reverse: 5′-CTT CCG TTT CCA AGG GCA C-3′; WSB2 forward: 5′- GCT GAG GTC ACT CCA CCA CAC-3′, reverse: 5′- CAG CAA AGC CCA TTG GTC AT-3′; GAPDH forward: 5′-AGG GCA TCC TGG GCT ACA C-3′, reverse: 5′-GCC AAA TTC GTT GTC ATA CCA G-3′.

**Transfection of siRNA and plasmids**

Cells were transfected with the following siRNA oligos or plasmids using Lipofectamine 2000. siCtrl: 5′-ATT GTA TGC GAT CGC AGA C-3′; siNOXA: 5′-GGT GCA CGT TTC ATC AAT T-3′; siASB1: 5′-AGC CAA CCT GAA TCT AGT GAA-3′; siASB2: 5′-CGA ACA TCG ACG CCT ATA T-3′; siASB3: 5′-GAA ATA TGG AGC CCA GAT A-3′; siASB4: 5′-GAC CAC AAT GCT ACA ATC AAC-3′; siASB5: 5′-GCT AAC TTG TTA CTG ATA TTG-3′; siASB6-1: 5′- GCA GAT CCA CAA TAC TGA GAA-3′; siASB6-2: 5′- GCC TTA ACA AGG TCC TTA TAT-3′; siASB7: 5′-GAC AGA CTC CTT TAC ACT TAT-3′; siASB8: 5′-GTC AGA GTC ATC AAC CTA ATA-3′; siASB9: 5′-GAG TGT GTC AAC TCT CTT ATA-3′; siASB10: 5′-GCA TGT CCT GAT GCC CGC AAT-3′; siASB11: 5′-GGA TAG CAG AAG AGA TCT ATG-3′; siASB12: 5′-TAT CCA GCT GTT AAT CGA TTT-3′; siASB13: 5′-CCC ACA AGT AGC ACT GAG TTT-3′; siASB14: 5′-TAC GGA TCT TGC TGC CAT TAA-3′; siASB15: 5′-GCT GAG GCT ATT GCT GAA TAA-3′; siASB16: 5′-GCT GAA ACA CTG CGC CAA CTT-3′; siASB17: 5′-ATG CTC CCA GAT GGA ATA TTT-3′; siASB18: 5′-CGT GGT GTT TGA GAT CAA TAA-3′; siSOCS1: 5′- CTA CCT GAG CTC CTT CCC CTT-3′; siSOCS2: 5′-GAA GGA ACT TTC TTG ATT A-3′; siSOCS3: 5′-CCA CCT GGA CTC CTA TGA GAA-3′; siSOCS4: 5′- GCA GTT GGA AAC ACC TCC TAA-3′; siSOCS5: 5′-TGC AAT TCC ACA AGC TAA TTG-3′; siSOCS6: 5′-CCT CAG GAC TAC ATT CAG TAT-3′; siSOCS7: 5′-CCT AAA CCT CTG ATC TCT TAT -3′; siCISH: 5′-CCT GCA CTG CTG ATA CCC GAA-3′; siSPSB1: 5′-GCT GCA TTC ATG GAA CAA CAA -3′; siSPSB2: 5′-GAA CTC TGG GCT ACG CTA TTG -3′; siSPSB3: 5′-TTC CCA GTG GAA CTG CCT TCT-3′; siSPSB4: 5′-GAG CCT CAA GTC AGT GGA GGT-3′; siWSB1: 5′-AGT TTC TCT CGT ATC GTA TTT-3′; siWSB2: 5′-CAC GGC TTC TTA CGA TAC CAA-3′; siRab40A: 5′-GGG TAT GGA TCG ATG GAT T-3′; siRab40B: 5′- CGG CAT TGA TCG ATG GAT TAA-3′; siRab40C: 5′-CCA GAA CTG CTC GCG GAG TAA-3′; siElongin A: 5′-CGC CAG TAG CAT CAG CTT TAA-3′; siMUF1: 5′-CCC AAC CAC CAG TTC TAT CTT-3′; siHSP90A: 5′-TAT GGC ATG ACA ACT ACT TTA-3′; siHSP90B: 5′-CGC ATG GAA GAA GTC GAT TAG-3′; 3×FLAG-tagged human WSB2, WSB2-∆SOCS, and 6×HIS-tagged Ub were subcloned into pLVX-IRES-Puro. FLAG-tagged human NOXA and NOXA (C3KR) were subcloned into pcDNA3.1.

***In* *vivo* ubiquitination assay**

HEK293 cells were transfected with indicated plasmids for 48 h, and then treated with 20 µM MG132 for 6 h. Subsequently, cells were lysed in a 6M guanidine denaturing solution and incubated with Ni-NTA agarose (1018244; Qiagen) for His-tagged protein purification, as described previously^1^.

**Flow cytometry**

Cells were transfected with indicated siRNA oligos for 48 h, and then analyzed by flow cytometry (CytoFLEX S; Beckman). Apoptosis was determined using an annexin V-FITC/propidium iodide (PI) apoptosis detection kit (C1062; Beyotime) according to the manufacturer’s instructions.

**Immunofluorescent staining**

Cells were fixed with 4% formaldehyde for 7 min, subsequently permeabilized with 0.5% Triton X-100 for 10 min. Afterward, cells were subjected to a 30-min blocking process and then incubated with anti-LC3B antibody (L7543; Sigma-Aldrich; 1:500) for 1 h, followed by staining with secondary antibodies labeled with Alexa Fluor 488 (Invitrogen; 1:500) for 1 h, and DAPI (Beyotime; 1:500) for an additional 30 min at room temperature. Finally, cells were photographed using a confocal fluorescence microscope (Nikon A1 Ti; Nikon).

**Cell Counting Kit 8 (CCK8)-based cell growth assay and clonogenic survival assay**

Cells were transfected with the specified siRNA oligos for 48 h, and then seeded in triplicate in 96-well plates. CCK8 assays (HY-K0301, MCE) were performed at various time points following the manufacturer's instructions. For the clonogenic assay, cells were plated in 60 mm dishes and cultured for 10–14 days before being stained with Coomassie Brilliant Blue. The dishes were photographed for colony counting (>50 cells in a colony).

**Statistical analysis**

The data from three independent biological experiments were presented as the mean ± SEM and statistically analyzed by GraphPad Prism 8. The significance of cell growth assays were determined by the two-way repeated-measures ANOVA analysis. Other statistical analyses were determined by Student’s *t*-tests. *p* < 0.05 was considered as statistically significant.

**References**

1. Cui D, Dai X, Shu J, et al. The cross talk of two family members of beta-TrCP in the regulation of cell autophagy and growth. *Cell Death Differ*. 2020;27(3):1119-1133.
